# Lightweight Safety Helmet Wearing Detection Algorithm Based on GSA-YOLO

**DOI:** 10.3390/s26072110

**Published:** 2026-03-28

**Authors:** Haodong Wang, Qiang Zhou, Zhiyuan Hao, Wentao Xiao, Luqing Yan

**Affiliations:** 1 Shandong Provincial Key Laboratory of New Power Distribution & Utilization Technology and Equipment, Shandong University of Technology, Zibo 255000, China; 2 School of Electrical and Electronic Engineering, Shandong University of Technology, Zibo 255000, China

**Keywords:** YOLO, safety helmet testing, I-ECA mechanism, GhostConv, WIoU

## Abstract

Electric power station confined spaces are high-risk and complex environments characterized by significant illumination variations. Whether safety helmets are properly worn directly affects the operational safety of workers in confined spaces. However, helmet detection in such environments faces several challenges, including drastic lighting changes and difficulties in small-object detection. Moreover, existing object detection models typically contain a large number of parameters, making real-time helmet detection difficult to deploy on field devices with limited computational resources. To address these issues, this paper proposes a lightweight safety helmet wearing detection algorithm named GSA-YOLO. To mitigate the effects of severe illumination variation and detail loss in confined spaces, a GCA-C2f module integrating GhostConv and the CBAM attention mechanism is embedded into the backbone network. This design reduces the number of parameters and computational cost while enhancing the model’s feature extraction capability under challenging lighting conditions. To improve detection performance for occluded targets, an improved efficient channel attention (I-ECA) mechanism is introduced into the neck structure, which suppresses irrelevant channel features and enhances occluded object detection accuracy. Furthermore, to alleviate missed detections of small objects and inaccurate localization under low-light conditions, a P2 detection branch is added to the head, and the WIoU loss function is adopted to dynamically adjust the weights of hard and easy samples, thereby improving small-object detection accuracy and localization robustness. A confined space helmet detection dataset containing 5000 images was constructed through on-site data collection for model training and validation. Experimental results demonstrate that the proposed GSA-YOLO achieves an mAP@0.5 of 91.2% on the self-built dataset with only 2.3 M parameters, outperforming the baseline model by 2.9% while reducing the parameter count by 23.6%. The experimental results verify that the proposed algorithm is suitable for environments with significant illumination variation and small-object detection challenges. It provides a lightweight and efficient solution for on-site helmet detection in confined space scenarios, thereby contributing to the reduction in industrial safety accidents.

## 1. Introduction

Typical electric power station confined space operations [1] include cable wells, transformer interiors, and fire-fighting water tanks. These environments present significant safety risks due to their enclosed structures and complex working conditions. In such scenarios, ensuring that workers wear safety helmets correctly is a critical requirement for operational safety. However, manual supervision is inefficient and unreliable, motivating the development of automated visual monitoring systems based on computer vision and deep learning techniques. It should be noted that the electric power station confined space scenarios considered in this study are limited to routine operation and maintenance or inspection environments, where arc discharge conditions do not occur.

Compared with open industrial environments, electric power station confined spaces exhibit several unique characteristics that make visual detection particularly challenging. First, illumination conditions are highly complex and variable. Worksites may be in complete darkness [2], low-light conditions [3,4], or partially exposed to strong illumination, leading to low image contrast and blurred visual details. Such conditions severely degrade feature extraction capability for detection models. Second, the spatial structure of confined spaces is narrow and densely populated with pipelines and electrical equipment. Workers often operate in constrained postures, causing safety helmets [5] to be partially or fully occluded, which leads to incomplete feature representation and potential misclassification. Third, image acquisition is frequently performed from long distances or from top-down and oblique perspectives, meaning that safety helmets occupy only a small proportion of pixels in the image [6] and are often embedded in complex backgrounds. These factors collectively increase missed detections and localization errors for conventional object detection models.

To address these challenges, researchers have explored various approaches to improve detection performance under complex environments. Existing studies mainly focus on three aspects: illumination-robust feature extraction, occlusion-aware detection, and small-object detection enhancement.

### 1.1. Illumination-Robust Feature Extraction Methods

Illumination variation is one of the primary factors affecting visual detection performance in confined environments. To mitigate this problem, some studies adopt image preprocessing methods to enhance low-light images before detection. For example, Sun Lin et al. [7] proposed a multi-weight fusion image enhancement approach to alleviate detection difficulties caused by uneven illumination. Although such preprocessing techniques can improve image visibility, they introduce additional computational overhead and may lack adaptability to dynamically changing lighting conditions.

Another research direction focuses on improving feature extraction through architectural modifications. Hou Gongyu et al. [8] reconstructed the backbone network of YOLOv5 using Ghost convolution to reduce computational complexity while incorporating channel attention to suppress background interference. Kang Chengyang et al. [9] proposed a multi-scale MELAN module for coal mine environments, integrating an enhanced attention mechanism into the ELAN structure of YOLOv7. Their MEMA module utilizes both global average pooling and max pooling to better preserve helmet detail information under low illumination.

In related domains, Qu et al. [2] proposed NoctuDroneNet for nighttime UAV imagery, demonstrating that channel-wise attention mechanisms can guide the network to focus on informative regions under low-contrast conditions. Similarly, Li et al. [4] introduced FE-YOLO, which incorporates Fourier-based feature enhancement to complement spatial features in low-light environments. These studies demonstrate that embedding lightweight convolution and attention mechanisms into backbone networks can effectively enhance feature representation under complex lighting conditions.

### 1.2. Occlusion Handling in Object Detection

Target occlusion is another critical challenge in confined environments, where workers frequently operate among dense equipment structures. Gu et al. [10] improved helmet detection accuracy under complex worker postures through posture-adaptive head localization. However, the cascaded architecture used in their approach introduces higher computational complexity, limiting real-time deployment.

Zhao Rui et al. [11] proposed an improved YOLOv5s algorithm to address occlusion and dense small-object detection problems, yet their method shows performance degradation under severe illumination variations. Wang et al. [12] enhanced multi-scale feature representation in YOLOv8-ADSC by incorporating attention mechanisms to better capture occluded regions, although the increased computational cost limits its lightweight deployment.

Hou Mingming et al. [13] introduced a Spatial Reconstruction Unit (SRU) into the backbone of YOLOv5 to reconstruct spatial features and reduce redundancy. Combined with efficient channel attention (ECA), this method effectively enhances the discrimination of useful features in occluded scenes. In another related study, Pradhan et al. [3] proposed a Graphically Residual Attentive Network (GRESIDAN), integrating residual learning with multi-headed attention to recover features lost due to occlusion. These works indicate that combining spatial and channel attention mechanisms can significantly improve the perception of partially occluded targets.

### 1.3. Small-Object Detection Enhancement

Small-object detection remains a fundamental challenge in object detection tasks, especially in confined environments where safety helmets occupy only a small proportion of image pixels [6]. The limited spatial information, combined with background interference and feature loss during downsampling, often leads to missed detections.

To improve small-object detection capability, researchers have explored multi-scale feature fusion and additional detection branches. Gao Lipeng et al. [14] designed a RepNMSC multi-scale feature extraction module and introduced a P2 detection head to reduce missed detections of small targets in confined environments. Yang Yongbo et al. [15] proposed YOLO-M3, a lightweight model optimized for mobile deployment, though its detection accuracy decreases significantly under drastic illumination changes.

Recent studies have also proposed several effective strategies. Cai et al. [16] reconstructed the C2f module using deformable convolution (DCNv2) and added a small-object detection head to enhance feature representation for small-scale targets in complex traffic scenes. Nie et al. [17] proposed DSOD-YOLO, which utilizes a dual-backbone architecture to enhance feature extraction for small objects and introduces a channel-scale adaptive module for feature selection. Zhang et al. [18] proposed MMAE-YOLOv8 for helmet detection in complex environments. Their method enhances small-object perception by introducing a C2f-MSEFA module for multi-scale edge feature aggregation, replacing the SPPF layer with a multi-scale attention convolution module, and designing an ASF-FS neck with an optimized P2 detection branch, which improves detection performance for small and partially occluded targets.

These studies demonstrate that enhancing shallow feature representation and introducing dedicated detection heads are effective strategies for improving small-object detection performance.

### 1.4. Research Gaps

Despite the significant progress achieved in recent studies, several challenges remain for helmet detection in electric power station confined spaces. First, most existing approaches address illumination variation, occlusion, and small-object detection independently, while these challenges often occur simultaneously in confined environments. Second, many methods improve detection robustness by introducing additional modules or complex architectures, which inevitably increases model parameters and computational cost, limiting deployment on resource-constrained edge devices. Third, current studies rarely focus on domain-specific optimization tailored to the characteristics of electric power station confined spaces, such as severe illumination fluctuations, narrow spatial structures, and long-distance image acquisition. Therefore, developing a lightweight yet robust detection framework specifically designed for such environments remains an important research problem. In this context, application-driven optimization of existing detection architectures is a practical and effective research direction for real-world deployment scenarios.

### 1.5. Overview of the Proposed Framework

To address the above challenges, this paper proposes a lightweight helmet detection framework named GSA-YOLO (Ghost–Small-object–Attention YOLO), developed based on the YOLOv8n architecture. Rather than introducing a fundamentally new detection paradigm, the proposed framework is designed as an application-driven lightweight optimization strategy tailored to the specific challenges of electric power station confined space environments. The name GSA reflects the three key design concepts of the proposed framework: the use of Ghost convolution for lightweight feature extraction, the enhancement of small-object detection capability, and the integration of attention mechanisms for improved feature representation. The framework integrates three key components. First, a GCA-C2f module is embedded into the backbone network to enhance feature extraction robustness under severe illumination variation while maintaining parameter efficiency through Ghost convolution. Second, an improved efficient channel attention (I-ECA) mechanism is introduced into the neck network to strengthen occlusion-aware feature representation without increasing model parameters. Third, a P2 detection head combined with the WIoU loss function is employed to improve localization accuracy for small and low-contrast targets. Through the coordinated design of these components, the proposed framework achieves robust detection performance in complex confined space environments while maintaining a lightweight architecture suitable for edge deployment.

### 1.6. Contributions

To address the above challenges, this study proposes a lightweight helmet-wearing detection algorithm named GSA-YOLO for electric power station confined space environments.

This work focuses on application-oriented optimization and structural adaptation of existing techniques rather than introducing entirely new model components. The main contributions of this paper are summarized as follows:

(1) Lightweight illumination-robust feature extraction via GCA-C2f.

To cope with severe illumination variations while maintaining parameter efficiency, we redesign the C2f Bottleneck by embedding Ghost convolution for lightweight feature generation and integrating a CBAM attention block to enhance feature representation under complex lighting conditions.

(2) Enhanced occlusion perception through the proposed I-ECA mechanism.

An improved efficient channel attention (I-ECA) mechanism is proposed to strengthen channel-wise feature interaction. By incorporating the GhostNet design philosophy, the proposed module enriches channel attention features without increasing parameters, enabling the network to better capture partially occluded helmet features.

(3) Small-target localization refinement using a P2 detection head and WIoU loss.

To improve detection accuracy for small and blurred helmets, a high-resolution P2 detection branch is introduced to preserve shallow spatial features, while the WIoU loss dynamically adjusts gradient contributions during training, improving localization stability and robustness.

Through the synergistic integration of these components, the proposed GSA-YOLO framework effectively addresses the challenges of illumination variation, occlusion, and small-object detection in electric power station confined spaces while maintaining a lightweight architecture suitable for real-time deployment.

## 2. Materials and Methods

### 2.1. GSA-YOLO Model

The proposed GSA-YOLO framework is developed based on the YOLOv8n baseline. Unlike existing studies that directly adopt the standard YOLOv8 architecture, the overall detection framework in this work is newly constructed for safety helmet detection in electric power station confined space environments. Rather than simply integrating off-the-shelf modules, our design philosophy emphasizes task-oriented integration and synergistic compatibility among lightweight components. Specifically, several improved modules are incorporated into the standard YOLOv8n architecture and reorganized to form a dedicated detection framework tailored for the challenges of confined space scenes.

**Figure 1:**
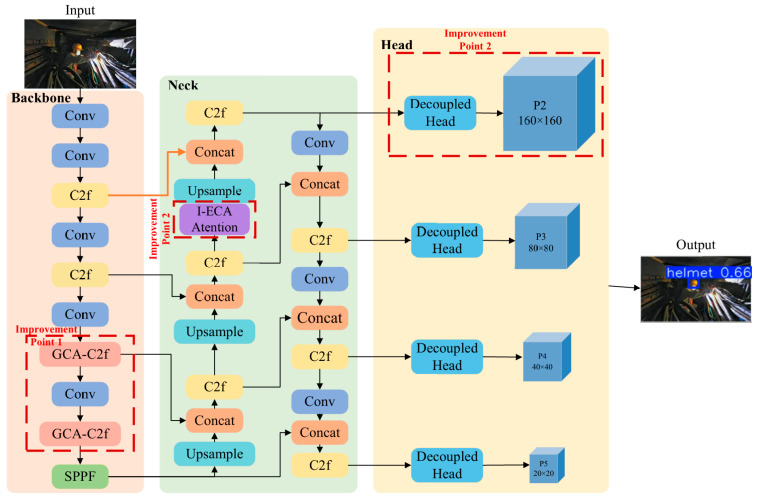
Overall architecture of the proposed GSA-YOLO framework for safety helmet detection in electric power station confined spaces. The framework is constructed based on YOLOv8n with three main improvements: GCA-C2f backbone modules, an I-ECA mechanism in the neck, and a P2 detection head with WIoU loss.

The overall architecture consists of three main components: the backbone, the neck, and the head, as illustrated in Figure 1. Figure 1 presents the overall framework proposed in this study, which is built upon the YOLOv8n backbone but redesigned through several structural modifications. As illustrated in Figure 1, the improvements are not independent; rather, they form a cohesive system where each component addresses specific challenges while complementing the others.

The backbone network is primarily composed of C2f modules and an SPPF module. In this study, two original C2f modules in YOLOv8n are replaced with the proposed GCA-C2f modules (Improvement 1 in Figure 1). The GCA-C2f module integrates the Ghost convolution module (GhostConv) [19] and the Convolutional Block Attention Module (CBAM) [20]. By combining lightweight Ghost feature generation with channel-spatial attention mechanisms, the proposed module effectively reduces model parameters while enhancing feature extraction capability under complex illumination conditions. This illumination-robust backbone provides cleaner feature maps for subsequent modules, laying the foundation for accurate detection in challenging confined space environments.

In the neck network, an improved efficient channel attention (I-ECA) mechanism is introduced (Improvement 2 in Figure 1). By strengthening channel feature responses and suppressing background interference, the I-ECA module improves detection accuracy for challenging targets, such as occluded or blurred helmets, in electric power station confined space environments. This module is integrated into the feature fusion stage of the neck network, enabling the model to better exploit multi-scale contextual information extracted by the backbone. Building upon the robust features extracted by the GCA-C2f backbone, the I-ECA mechanism further refines channel-wise representations to better handle occlusion scenarios.

In the head network, a P2 detection branch is added (Improvement 3 in Figure 1) to enhance the model’s sensitivity to small-scale objects, thereby effectively reducing missed detections. The introduction of the P2 detection layer allows the network to preserve higher-resolution spatial information, which is particularly beneficial for detecting small helmets in surveillance images captured within confined spaces. Furthermore, regarding the loss function design, the original model adopts the Complete Intersection over Union (CIoU) loss. However, CIoU has limitations in bounding box size adjustment and insufficient consideration of non-overlapping spatial relationships. Therefore, the WIoU loss is employed to replace CIoU in this study. The WIoU loss dynamically adjusts the contribution of different training samples, thereby improving localization robustness for blurred targets under low-light conditions. Together, the P2 head and WIoU loss jointly refine the localization of small and blurred targets, addressing the coupled challenges of scale variation and low-light degradation.

The overall architecture of the improved YOLOv8n-based GSA-YOLO detection framework proposed in this work is shown in Figure 1.

#### 2.1.1. GCA-C2f Module

The C2f module in the original YOLOv8 architecture is designed to enhance feature reuse and gradient propagation through a split–transform–concatenate structure. Specifically, the input feature map is first processed by a convolution layer and then split into multiple branches. These branches pass through a series of Bottleneck layers before being concatenated to generate the final feature representation. Although this structure improves feature aggregation capability, the Bottleneck blocks rely on standard convolution operations, which introduce redundant parameters and computational overhead.

**Figure 2:**
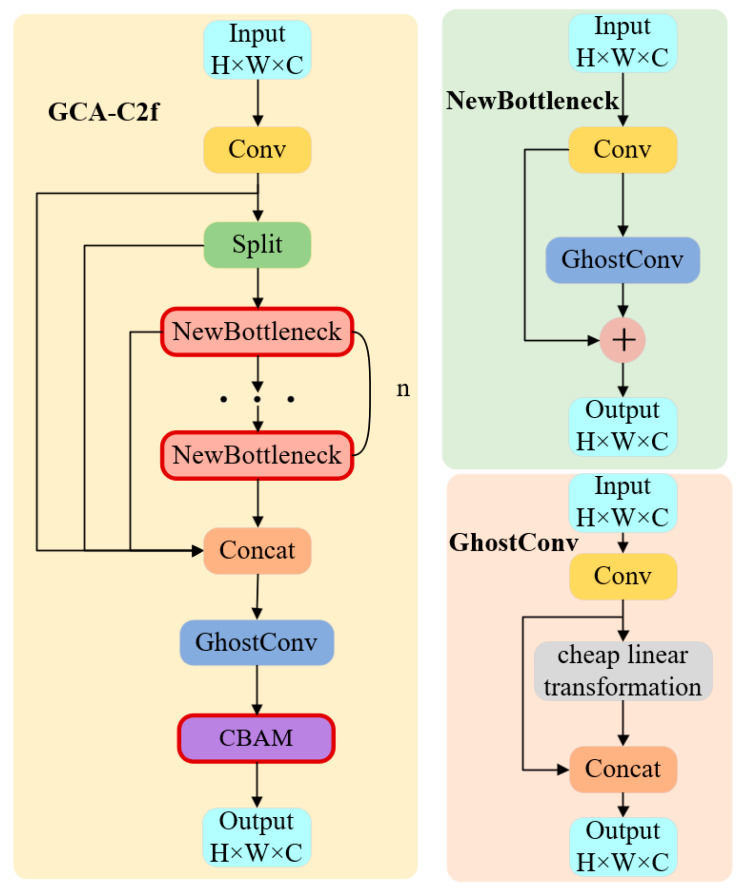
Structure of the proposed GCA-C2f module and its internal components.

To address these limitations, this study proposes a GCA-C2f module, which integrates Ghost convolution and an attention mechanism into the original C2f architecture. The structure of the proposed module is illustrated in Figure 2.

Where *H*, *W* and *C* denote the height, width, and number of channels, the input first passes through a convolution layer for initial feature extraction. The resulting feature map is then split into multiple branches, where a subset of features is directly preserved while the remaining features are fed into several stacked NewBottleneck blocks.

Unlike the original Bottleneck structure, the proposed NewBottleneck replaces the standard convolution operation with GhostConv, which generates intrinsic feature maps through standard convolution and then produces additional feature maps using inexpensive linear transformations. This design effectively reduces redundant computation while maintaining feature representation capability. The structure of the NewBottleneck block is shown in Figure 2.

After passing through multiple NewBottleneck blocks, all branch features are concatenated along the channel dimension:

(1) $$F_{concat}=Concat(F_{1},F_{2},\ldots ,F_{n})$$

To further enhance feature extraction efficiency, an additional GhostConv layer is introduced after the concatenation operation to refine the fused feature representation while maintaining lightweight computation.

Finally, a Convolutional Block Attention Module (CBAM) is appended to the end of the module to adaptively recalibrate feature responses. The attention refinement process can be expressed as

(2) $$F^{\prime }=M_{c}(F)⊗F$$

(3) $$F^{\prime \prime }=M_{s}(F^{\prime })⊗F^{\prime }$$

where *M_c_*(⋅) and *M_s_*(⋅) represent the channel attention and spatial attention functions, respectively, and ⊗ denotes element-wise multiplication.

Compared with the original C2f module, the proposed GCA-C2f introduces three key structural modifications:

(1) The Bottleneck blocks are redesigned as NewBottleneck modules using GhostConv, reducing computational redundancy.
(2) A GhostConv layer is added after feature concatenation to improve feature fusion efficiency.
(3) A CBAM attention module is introduced at the end of the block to enhance the model’s ability to focus on important spatial and channel information.

Through these modifications, the GCA-C2f module simultaneously improves feature extraction capability and reduces computational cost, making it more suitable for lightweight object detection in complex electric power station confined space environments.

#### 2.1.2. Improved ECA Mechanism

The efficient channel attention (ECA) mechanism is a lightweight channel attention method that improves feature representation without introducing dimensionality reduction. Unlike traditional channel attention mechanisms such as SE blocks, ECA captures local cross-channel interactions through a lightweight one-dimensional convolution, which significantly reduces computational complexity.

Given an input feature map *X* ∈ *R^H^*^×*W*×*C*^, where *H*, *W*, and *C* denote the height, width, and number of channels respectively, global average pooling (GAP) is first applied to obtain a channel descriptor *z* ∈ *R*^1×1×*C*^. A one-dimensional convolution is then used to model local channel interaction. The convolution kernel size *k* is adaptively determined according to the channel dimension *C*:

(4) $$k=\psi (C)={\left|\frac{\log_{2}(C)}{a}+b\right|}_{odd}$$

where *a* and *b* are hyperparameters controlling the mapping between channel dimension and convolution kernel size *k*.

Although the original ECA mechanism is efficient, the channel interaction is generated through a single convolution operation, which limits its feature representation capability in scenarios with occlusion and low-contrast targets, such as electric power station confined spaces.

**Figure 3:**
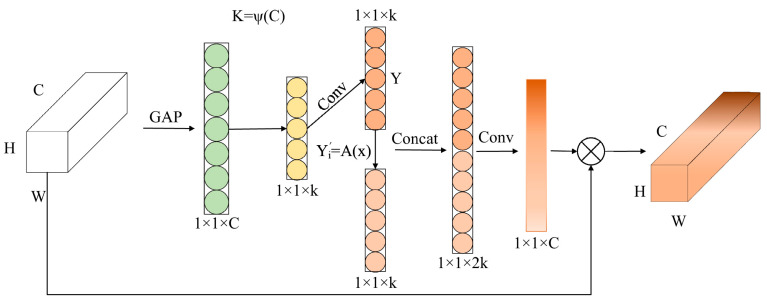
Structure of the I-ECA module.

To address this limitation, this study proposes an improved efficient channel attention (I-ECA) mechanism. The key idea of I-ECA is to expand channel interaction features before generating channel attention, thereby enriching channel information while maintaining a lightweight structure. The structure of the proposed I-ECA module is illustrated in Figure 3.

Specifically, after obtaining the intermediate feature vector *f* ∈ *R*^1×1×*k*^ through the convolution operation (corresponding to the 1 × 1×k feature shown in Figure 3), the module introduces a lightweight feature expansion strategy. First, a standard 1 × 1 convolution is applied to generate intrinsic channel features: *Y* ∈ *R*^1×1×*k*^.

Where *Y* = [*y*_1_, *y*_2_, …, *y_k_*], corresponding to the upper branch in Figure 3.

Then, a parallel branch is introduced to generate complementary channel features through a linear transformation, as illustrated in Figure 3:

(5) $$Y_{i}^{\prime }=A(y_{i}),\;i=1,2,\ldots ,k$$

where *Y*′ ∈ *R*^1×1×*k*^ denotes the generated complementary feature map (lower branch in Figure 3), and A(⋅) represents a lightweight channel transformation operation.

The intrinsic features and the generated complementary features are concatenated [21] to form an expanded feature representation, *F*_concat_ ∈ *R*^1×1×2*k*^. *F*_concat_ = [*Y*, *Y*′], which corresponds to the 1 × 1 × 2 k feature after the Concat operation in Figure 3.

Finally, a 1 × 1 convolution is used to map the expanded feature representation back to the original channel dimension:

(6) $$A=\sigma (WF_{concat}),\;A\in R^{1\times 1\times C}$$

where *W* denotes the convolution weight matrix and σ(⋅) represents the activation function. This operation realizes the mapping from 1 × 1 × 2 *k* to 1 × 1 × *C*, as illustrated in Figure 3. The obtained attention vector is multiplied with the input feature map in a channel-wise manner:

(7) $$X^{\prime }=X⊗A$$

where ⊗ denotes channel-wise multiplication.

Compared with the standard ECA mechanism, the proposed I-ECA module introduces a dual-branch feature expansion structure in the channel interaction stage, which enriches channel information representation before generating channel attention weights.

From a feature representation perspective, the original ECA mechanism models channel interaction through a single convolution operation, which limits its ability to capture diverse channel dependencies, especially under complex conditions such as occlusion and low contrast. In contrast, the proposed dual-branch structure generates complementary channel responses by combining intrinsic features and transformed features, effectively increasing channel feature diversity without introducing additional parameters. This expanded representation enables the attention mechanism to better distinguish informative channels from redundant background features, thereby improving robustness to partial occlusion and feature ambiguity. This design improves the model’s ability to capture discriminative features of partially occluded helmets while maintaining the lightweight characteristics required for edge deployment.

By integrating the I-ECA mechanism into the neck of the YOLOv8n model, the feature extraction capability for helmet regions of electric power station workers is significantly enhanced. Moreover, the model’s adaptability to severe illumination variations in confined space environments is improved without increasing the overall parameter size of YOLOv8n.

#### 2.1.3. P2 Detection Branch

Due to the structural complexity of electric power station confined space environments, in some operation scenarios the distance between workers and monitoring equipment is relatively large. Consequently, safety helmets often appear as small-scale targets in captured images. In the original YOLOv8n architecture, input video frames are first processed by the backbone network for feature extraction, followed by feature fusion in the neck network. The detection head then outputs three feature maps at different scales, with spatial resolutions of 20 × 20, 40 × 40 and 80 × 80, respectively. However, experimental results indicate that the original YOLOv8n model exhibits limited performance in small-object detection tasks. To address this issue, an additional P2-level detection head with an output resolution of 160 × 160 is introduced into the head network. The higher-resolution feature map preserves more fine-grained spatial information, thereby enhancing the model’s sensitivity to small-scale targets and effectively reducing missed detections in confined space scenarios.

**Figure 4:**
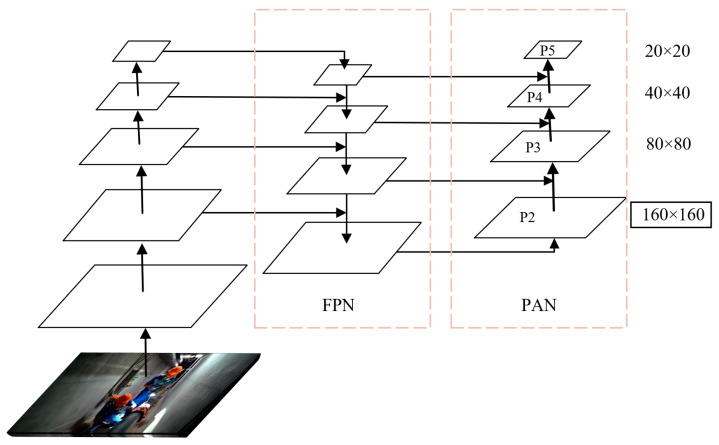
Small-object detection head.

The feature extraction and fusion process after introducing the P2 detection branch is illustrated in Figure 4. In the original detection head, the three output feature maps are generated from the input image (640 × 640 pixels) after downsampling by factors of 8, 16 and 32, respectively. However, after multiple convolutional operations, fine-grained spatial details are inevitably lost in deeper feature maps. This loss of low-level information adversely affects the detection performance of small-scale targets. To address this issue, the input of the newly added small-object detection head is constructed by concatenating two feature maps. One branch is derived from the second-stage feature map of the backbone network, which retains rich low-level spatial details. The other branch comes from the existing feature map in the neck network, which contains higher-level semantic information. By fusing shallow spatial features with deeper semantic features, the proposed structure effectively preserves detailed information while maintaining contextual representation. The fused feature map ultimately generates an output with a spatial resolution of 160 × 160 pixels. The detection head operating at this higher resolution focuses more on fine-grained image details and utilizes a relatively smaller receptive field, thereby significantly enhancing the model’s performance in small-object detection tasks.

#### 2.1.4. Improvement of the Loss Function

The YOLOv8n model adopts the Complete Intersection over Union (CIoU) loss [22,23] as its bounding box regression loss. Although CIoU considers the overlap area, center distance, and aspect ratio consistency between the predicted box and the ground-truth box, its strict penalty mechanism may excessively amplify regression errors when low-quality samples are present. In the collected confined space dataset, small targets under low-illumination conditions often produce unstable regression signals, which may hinder model convergence and weaken overall detection performance.

To address this issue, the CIoU loss is replaced with the Wise Intersection over Union loss. The WIoU loss can be expressed as:

(8) $$L_{IoU}=1-IoU=1-\frac{W_{i}H_{i}}{wh+w_{gt}h_{gt}-W_{i}H_{i}}$$

(9) $$R_{WIOU}=exp\left(\frac{{\left(x-x_{gt}\right)}^{2}+{\left(y-y_{gt}\right)}^{2}}{{\left(W_{g}^{2}+H_{g}^{2}\right)}^{*}}\right)$$

(10) $$L_{WIOUv1}=R_{WIOU}L_{IoU}$$

where *L_IoU_* represents the IoU-based regression term, and *R_WIoU_* denotes the dynamic focusing coefficient. In the formulation, *W_g_* and *H_g_* denote the width and height of the minimum enclosing box covering both the predicted box and the ground-truth box, respectively. Wi and *H_i_* represent the width and height of the predicted box, while *w_gt_* and *h_gt_* denote the width and height of the ground-truth box. The superscript “*” indicates a gradient-adjusted term designed to suppress factors that may hinder model convergence. When the overlap between the predicted box and the ground-truth box is large, the *L_IoU_* term focuses more on the center distance between the two boxes. The coefficient *R_WIoU_* further computes the center distance between the predicted and ground-truth boxes and applies a scale-aware weighting strategy based on the size of the target box. This design strengthens the sensitivity to localization discrepancies while preventing excessive penalties on low-quality samples. By introducing the dynamic weighting mechanism, WIoU effectively balances regression difficulty and sample quality, improves convergence stability, and enhances localization robustness for small and low-contrast targets in confined space environments.

Compared with Wise-IoU v1, Wise-IoU v3 introduces a proportional factor β and a scaling factor γ into the loss function, which enables the model to more flexibly adjust the influence of different targets during training. The formula is expressed as:

(11) $$\beta =\frac{L_{IoU}^{v}}{{\bar{L}}_{IoU}}\in [0,+\infty )$$

(12) $$L_{WIOUv3}=rL_{WIOUv1}$$

(13) $$r=\frac{\beta }{\delta \alpha^{\beta -\delta }}$$

where $L_{IoU}^{v}$ is the bounding box loss, and ${\bar{L}}_{IoU}$ is the average bounding box loss. The ratio between them, *β*, changes continuously with the variation in the bounding box loss during training, thereby continuously adjusting the influence of the target on the model training loss and further improving the detection performance of the model.

The WIoU loss function adaptively adjusts the gradient weights through a dynamic focusing mechanism, enabling the model to pay more attention to the detection of hard targets during training [24]. This dynamic optimization characteristic is particularly important in power station confined space scenarios where there are large differences in target scales and a high similarity between targets and the background.

**Figure 5:**
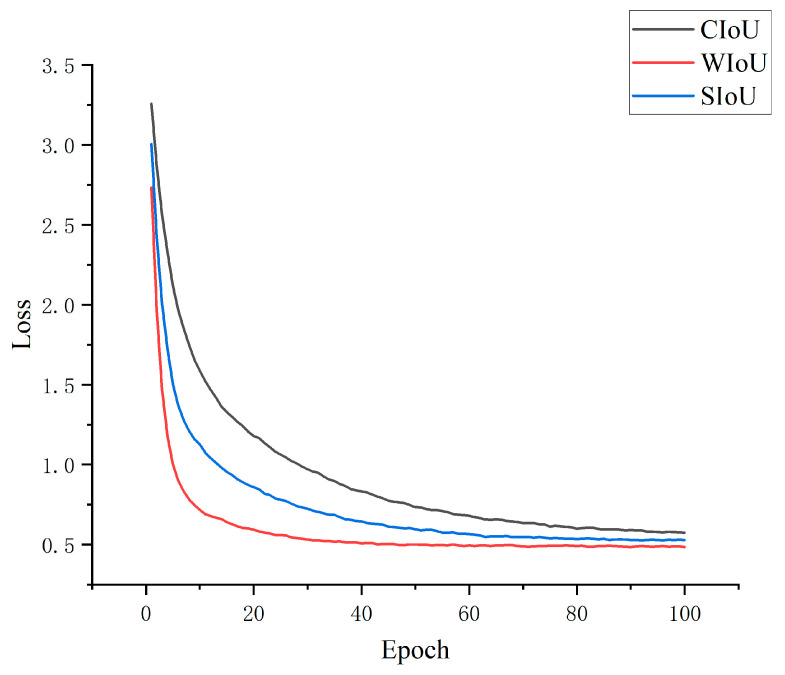
Comparison of loss functions.

To verify the effectiveness of the WIoU loss function, this study compares the WIoU loss with the original CIoU loss and the SIoU loss in the model after introducing the GCA-C2f module and adding the P2 small-object detection layer. As shown in Figure 5, when the model adopts the WIoU loss function, it can identify target objects more accurately and rapidly while providing high-confidence prediction results. In addition, the model converges with fewer training iterations.

## 3. Results

### 3.1. Experimental Environment

This experiment was conducted on the Windows 11 operating system. The hardware configuration and software environment are as follows:

(1) CPU: Intel Core i5-12600KF processor.
(2) GPU: NVIDIA GeForce RTX 4070 SUPER with 12 GB of video memory.
(3) Deep Learning Framework: PyTorch 2.4.1.
(4) Programming Language: Python 3.12.5.
(5) CUDA Version: 12.1.

The baseline model used in this study is YOLOv8n (version 8.3.40.1), specifically the official implementation released by Ultralytics. All experiments, including baseline comparisons and ablation studies, were conducted using the same software environment and hardware platform to ensure fair and consistent evaluation.

The training hyperparameters were uniformly configured for all models in the comparative experiments as shown in Table 1.

To ensure a fair comparison, all baseline models (including SSD, YOLOv3-tiny, YOLOv5n, YOLOv7-tiny, YOLOv8n, YOLOv10n, YOLOv11n, YOLOv13n, Improve-YOLOv5 [11], GCW-YOLOv8n [25], CGALS-YOLO [26] and YOLOv11n-DDH [27]) were retrained from scratch using the identical training settings listed above. No pre-trained weights were used for any of the compared models, and all experiments were conducted on the same training/validation/test split of the self-constructed dataset. This consistent experimental setup guarantees that the performance improvements reported in this paper are attributable solely to the proposed architectural modifications rather than differences in training protocols.

### 3.2. Datasets and Evaluation Metrics

#### 3.2.1. Experimental Data

**Figure 6:**
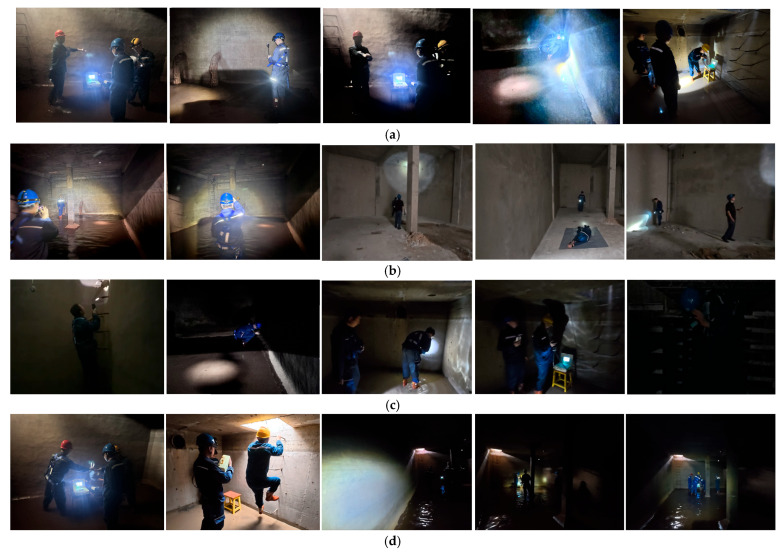
Partial visualization results of detection outputs. (**a**) Objects with significant changes in illumination, (**b**) small object, (**c**) low-illumination object, (**d**) occluded object.

Through on-site field investigation, image data were collected from typical power station confined space scenarios such as cable trenches, fire water tanks, and accident oil pools. The data collection process was conducted under real operational conditions to ensure the dataset reflects the actual challenges of confined space environments. A total of 5000 images were captured, and some representative samples are shown in Figure 6.

(1) Data Collection Conditions:

Images were acquired using a DJI Osmo Action 5 Pro action camera (SZ DJI Technology Co., Ltd., Shenzhen, China) mounted on tripods or handheld by operators to simulate both fixed surveillance and mobile inspection scenarios. The camera was positioned at distances ranging from 2 to 8 m from the workers, capturing footage from top-down and oblique angles to reflect realistic monitoring perspectives in confined spaces. To cover diverse illumination conditions, recordings were performed at different times of the day (morning, afternoon, evening) and under various lighting scenarios, including: (1) natural illumination with adequate lighting, (2) low-light conditions where only headlamps and flashlights were available, (3) complete darkness simulated by turning off all lights, and (4) scenes with mixed lighting where strong external light partially illuminated dark areas. The Osmo Action 5 Pro’s high dynamic range (HDR) mode was enabled to better capture details in challenging lighting conditions, ensuring that the dataset preserves critical visual information even under extreme illumination variations. This variety ensures that the dataset encompasses the full spectrum of illumination variations encountered in real-world confined space operations.

(2) Dataset Composition and Class Distribution:

The dataset covers various working environments, including cable wells, fire water tanks, cable trenches, and accident oil pools. It includes targets with significant illumination variations, small-scale targets (with pixel area less than 32 × 32), targets in low-illumination conditions, and targets with occlusion. As shown in Table 2, the dataset consists of 5000 images in total, including 4000 images for training and 1000 images for testing. In terms of category distribution, it contains 1193 images with significant illumination variation, 909 images of small targets, 1604 images under low-illumination conditions, and 1294 images with occluded targets. The detailed data distribution is presented in Table 2.

(3) Handling of Class Imbalance:

As shown in Table 2, the four scenario categories exhibit some degree of class imbalance (e.g., low-illumination targets account for 32.1% of the dataset, while small targets account for only 18.2%). To mitigate the potential impact of this imbalance on model training, we adopted the following strategies: (1) during training, we applied a combination of data augmentation techniques including random flipping, rotation, scaling, and color jittering to effectively increase the diversity and representation of underrepresented categories and (2) the loss function used in our final model (WIoU) incorporates a dynamic focusing mechanism that adaptively adjusts the contribution of hard and easy samples, which inherently helps balance the influence of different categories during training. Experimental results in Section 3.3 demonstrate that our proposed GSA-YOLO achieves consistent performance improvements across all four categories, indicating that the imbalance issue has been effectively mitigated.

(4) Annotation Procedure and Quality Control:

All images were annotated using LabelImg by a team of three researchers with prior experience in object detection annotation and knowledge of electric power station safety standards. Two categories were defined: “Helmet” (worker wearing a safety helmet) and “Head” (worker not wearing a safety helmet). The annotation process followed strict guidelines: (1) bounding boxes were drawn tightly around the helmet or head region, including only the target object; (2) for partially occluded targets, annotators were instructed to include only the visible portion within the bounding box; and (3) targets smaller than 10 × 10 pixels or with more than 80% occlusion were marked as “ignore” regions and excluded from training to avoid introducing noise.

To ensure annotation quality, a two-stage verification process was implemented:

Stage 1: After initial annotation by one researcher, another researcher independently reviewed all annotations and flagged any inconsistencies or errors. Disagreements were resolved through discussion.

Stage 2: A senior researcher with expertise in electric power station safety operations randomly sampled 20% of the annotated images for final verification. The inter-annotator agreement rate reached 96.3%, confirming the reliability of the annotations.

After annotation, YOLO-format label files were automatically generated. The dataset was then divided into training and testing sets at a ratio of 8:2 for model training and evaluation.

(5) Dataset Availability:

The dataset constructed in this study is currently not publicly available due to confidentiality agreements with the partner power station companies involved in data collection. However, portions of the dataset may be made available upon reasonable request to the corresponding author, subject to institutional approval and data usage agreements. We are also exploring options to release an anonymized version of the dataset in the future to facilitate further research in this domain.

#### 3.2.2. Evaluation Metrics

To accurately evaluate the performance of the model, five metrics were used for comparison, including precision (P), recall (R), mean Average Precision (mAP), number of parameters (Params), and the loss function.

Precision measures the proportion of samples that are actually positive among all samples predicted as positive, reflecting the accuracy of the model in predicting positive samples. Recall evaluates the proportion of samples correctly predicted as positive among all actual positive samples, reflecting the model’s ability to identify positive samples. Recall represents the number of positive samples that the model can correctly detect. The calculation formula is as follows:

(14) $$Precision=\frac{TP}{TP+FP}$$

(15) $$\mathrm{Recall}=\frac{TP}{TP+FN}$$

Among them, true positive (TP) represents the number of samples correctly predicted as positive by the model. False positive (FP) represents the number of samples in which negative samples are incorrectly predicted as positive by the model. False negative (FN) represents the number of samples in which positive samples are incorrectly predicted as negative by the model.

Average Precision (AP) is a metric used to evaluate model performance by calculating the area under the curve of the relationship between precision and recall. In object detection tasks, AP is usually calculated separately for each category, and then the average of all AP values is taken to obtain the mean Average Precision (mAP). When the Intersection over Union (IoU) threshold is set to 0.5, mAP is denoted as mAP@0.5. A higher mAP value indicates higher average detection accuracy and better model performance. The calculation formula is as follows:

(16) $$AP=\int_{0}^{1}P(R)dR$$

(17) $$mAP=\frac{1}{C}\sum_{i=1}^{C}AP_{i}$$

Among variables, C represents the number of data categories.

The number of model parameters (Params) refers to the total number of trainable parameters in the model, which is an important indicator for measuring the complexity and scale of the model. The larger the number of parameters, the higher the complexity of the model, and the more storage space is required to store these parameters. In addition, a larger number of parameters also increases the demand for computational resources, including memory and computing capacity.

GFLOPs (Giga Floating Point Operations) represents the number of floating-point operations required during a single forward inference of the model, which reflects the computational complexity of the network. A lower GFLOP value generally indicates a more computationally efficient model.

FPS (Frames Per Second) denotes the number of images that the model can process per second during inference, which is an important metric for evaluating real-time performance. A higher FPS indicates faster inference speed and better suitability for real-time applications. It should be noted that the reported FPS values in this study are measured on a high-performance desktop GPU, and thus the suitability for edge deployment is inferred from computational efficiency metrics rather than directly validated on actual edge devices.

Therefore, Params, GFLOPs, and FPS are jointly used to evaluate the model complexity, computational efficiency, and real-time inference capability.

#### 3.2.3. Improved Algorithm Experiments

**Figure 7:**
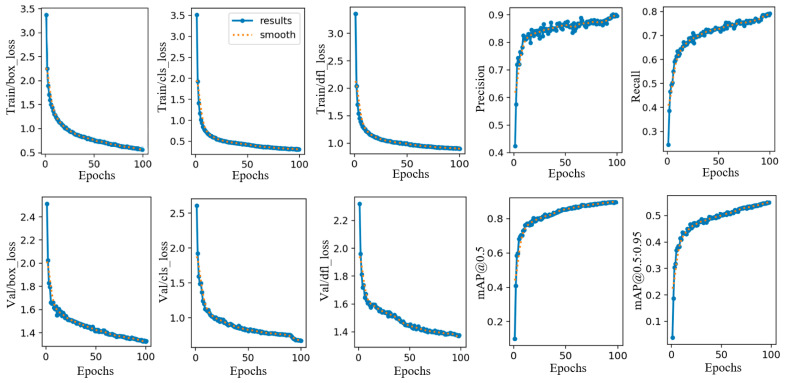
Training and validation loss and performance metric curves of GSA-YOLO on a self-built safety helmet dataset.

To further validate the effectiveness of the improved model, experiments were conducted on the self-constructed dataset. The training and validation curves of box_loss, cls_loss, and dfl_loss, as well as the convergence curves of performance metrics including precision, recall, mAP@0.5, and mAP@0.5:0.95, are shown in Figure 7. The horizontal axis represents the number of training epochs, with a total of 100 epochs. As illustrated in Figure 7, all loss curves gradually decrease and approach zero after approximately 70 epochs, indicating that the model reaches convergence. Ultimately, the box_loss stabilizes at approximately 0.21.

### 3.3. Ablation Experiments

To validate the effectiveness of each proposed component, ablation experiments were conducted, and the results are presented in Table 3.

Replacing the C2f module in the backbone with the proposed GCA-C2f reduces the number of parameters from 3.01 M to 2.03 M, corresponding to approximately 67.5% of the baseline YOLOv8n model. Meanwhile, the computational complexity decreases from 8.2 GFLOPs to 6.7 GFLOPs, and the inference speed increases from 89.4 FPS to 101.6 FPS, indicating improved computational efficiency. Although precision and recall slightly decrease by 0.5% and 0.3%, respectively, the reductions in mAP@0.5 and mAP@0.5–0.95 are minimal (0.7% and 0.1%), demonstrating that the lightweight optimization is achieved with negligible performance degradation.

Embedding the I-ECA mechanism into the neck network improves feature representation capability. Compared with the baseline model, precision and recall increase by 0.1% and 0.6%, respectively, while mAP@0.5 and mAP@0.5–0.95 improve by 0.8% and 1.7%. The computational complexity slightly increases to 8.4 GFLOPs, and the inference speed decreases marginally to 88.6 FPS, which is expected due to the additional attention computation. Nevertheless, the overall performance gain indicates that the proposed attention mechanism effectively enhances feature discrimination.

Adding the P2 small-object detection branch significantly improves the model’s ability to detect small targets. Compared with the baseline model, precision increases by 0.3%, and mAP@0.5–0.95 increases by 1.5%. However, the additional detection layer introduces extra computation, increasing the complexity to 9.2 GFLOPs and reducing the inference speed to 83.7 FPS.

Replacing the original loss function with WIoU improves precision and recall by 0.4% and 1.5%, respectively, and increases mAP@0.5–0.95 by 1.4%. Since the loss function only affects the training process, the number of parameters, GFLOPs, and inference speed remain unchanged compared with the baseline model. In addition, the model converges earlier during training, indicating improved optimization stability.

### 3.4. Comparative Experiments

**Figure 8:**
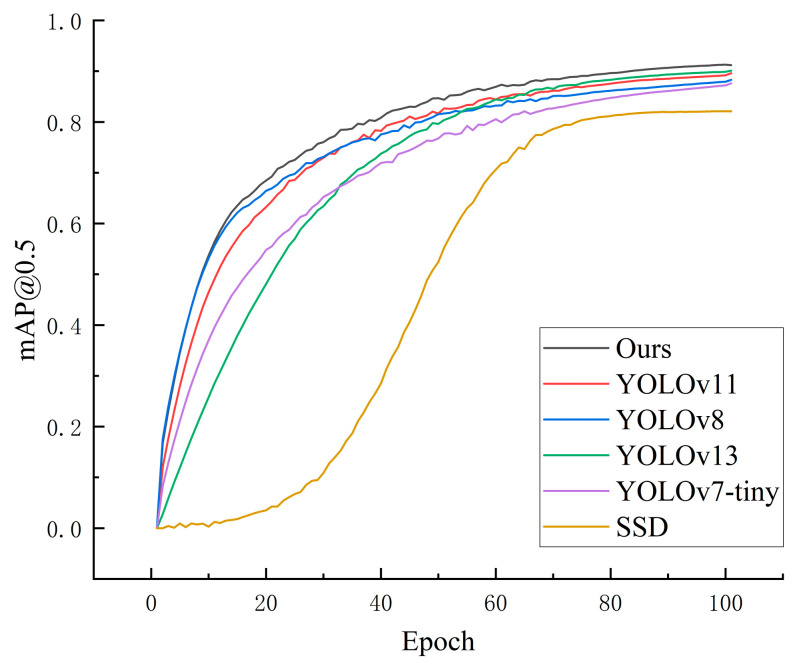
Comparison of mAP@0.5 curves for different models.

To more comprehensively validate the effectiveness of the proposed GSA-YOLO model, it was compared with several mainstream object detection models as well as existing studies specifically designed for safety helmet detection. The test dataset was collected from cable wells, fire water tanks, accident oil pools, and cable trenches. Under the same dataset, the experimental results of SSD, YOLOv3-tiny, YOLOv5n, YOLOv7-tiny, YOLOv8n, YOLOv10n, YOLOv11n, YOLOv13n, Improve-YOLOv5, GCW-YOLOv8n, and the proposed GSA-YOLO are presented in Table 4. Figure 8 shows the comparison of the mAP@0.5 curves between the proposed model and several representative detectors. The experimental results demonstrate that the proposed GSA-YOLO achieves the best overall performance among all compared models in terms of detection accuracy, efficiency, and lightweight characteristics.

As shown in Table 4, the proposed GSA-YOLO achieves the highest detection accuracy among all compared models, reaching 91.2% mAP@0.5 and 57.1% mAP@0.5–0.95, which represent improvements of 2.9% and 2.7%, respectively, over the baseline YOLOv8n model. At the same time, the parameter count is reduced from 3.01 × 10^6^ to 2.30 × 10^6^, indicating that the proposed improvements effectively enhance accuracy while maintaining a lightweight architecture.

Compared with other lightweight YOLO variants, the proposed model also demonstrates superior performance. For example, GSA-YOLO outperforms YOLOv3-tiny, YOLOv5n, YOLOv7-tiny, YOLOv10n, YOLOv11n, and YOLOv13n in terms of mAP@0.5 by 5.9%, 4.1%, 3.6%, 4.1%, 1.6%, and 1.1%, respectively. In addition, the proposed method maintains competitive computational complexity with 7.8 GFLOPs while achieving a high inference speed of 92.6 FPS, demonstrating a favorable balance between detection accuracy and real-time performance.

Furthermore, the proposed method is compared with recent studies specifically designed for safety helmet detection. The Improve-YOLOv5 algorithm enhances YOLOv5 by introducing modules such as DenseBlock, SE-Net attention, and improved data augmentation to improve feature extraction and small-object detection capability. Although this method improves the detection accuracy of YOLOv5-based models, it introduces additional network complexity and results in a relatively large parameter size (7.51 × 10^6^), which may limit its deployment on resource-constrained devices.

Similarly, the GCW-YOLOv8n model introduces a GS-C2f module with GhostConv and SE attention mechanisms, along with CBAM attention and the WIoU loss function to improve feature extraction and detection performance while reducing model complexity. Compared with this method, the proposed GSA-YOLO further improves detection accuracy and achieves higher mAP values while maintaining comparable computational complexity and real-time inference speed.

Overall, the experimental results indicate that the proposed GSA-YOLO model not only surpasses general object detection models but also outperforms existing helmet detection algorithms in terms of detection accuracy, parameter efficiency, and inference speed. These advantages make the proposed method more suitable for real-time safety helmet monitoring in power station confined space environments where both accuracy and computational efficiency are critical. However, it should be noted that this conclusion is based on experimental results obtained on a desktop GPU platform, and the deployment capability on edge devices is inferred rather than directly validated.

### 3.5. Generalization Validation on a Public Dataset

Model generalization refers to the ability of a machine learning model to perform well on previously unseen data, which is crucial for evaluating whether the model can be reliably applied in real-world scenarios. Strong generalization capability helps improve detection accuracy, prevent overfitting, and ensure stable performance during practical deployment.

To further validate the generalization ability of the proposed GSA-YOLO model, experiments were conducted on the publicly available safety helmet dataset. This dataset contains 5000 high-quality annotated images, which are divided into a training set (4000 images, 80%), a validation set (500 images, 10%), and a test set (500 images, 10%).

As shown in Table 5, the proposed GSA-YOLO model was compared with the baseline YOLOv8n model on a public dataset. The experimental results demonstrate that GSA-YOLO achieves superior overall performance. Specifically, the proposed method attains 88.1% mAP@0.5, 55.1% mAP@0.5–0.95, 88.6% precision, and 79.5% recall, outperforming YOLOv8n by 3.4%, 3.8%, 2.1%, and 1.2%, respectively.

In addition, the proposed model reduces the number of parameters from 3.12 M to 2.36 M and decreases computational complexity from 7.9 GFLOPs to 7.6 GFLOPs, while the inference speed increases from 91.5 FPS to 93.7 FPS.

These results indicate that the proposed method achieves a better balance between detection accuracy and computational efficiency. Moreover, the consistent performance improvements on the public dataset further demonstrate the strong generalization capability of the proposed model.

**Figure 9:**
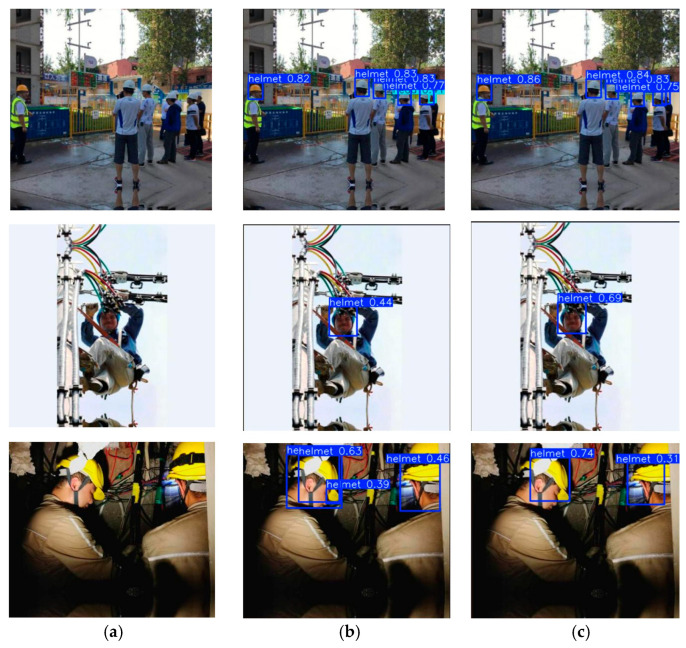
Comparison of detection results on a public dataset: (**a**) artwork, (**b**) YOLOv8n, (**c**) proposed algorithm.

The improved YOLOv8n algorithm proposed in this paper demonstrates better detection performance on the public dataset compared with the baseline YOLOv8n model, as shown in Figure 9. The original YOLOv8n model tends to produce false detections when dealing with dense targets and objects under low-illumination conditions.

### 3.6. Comparative Analysis of Detection Performance

**Figure 10:**
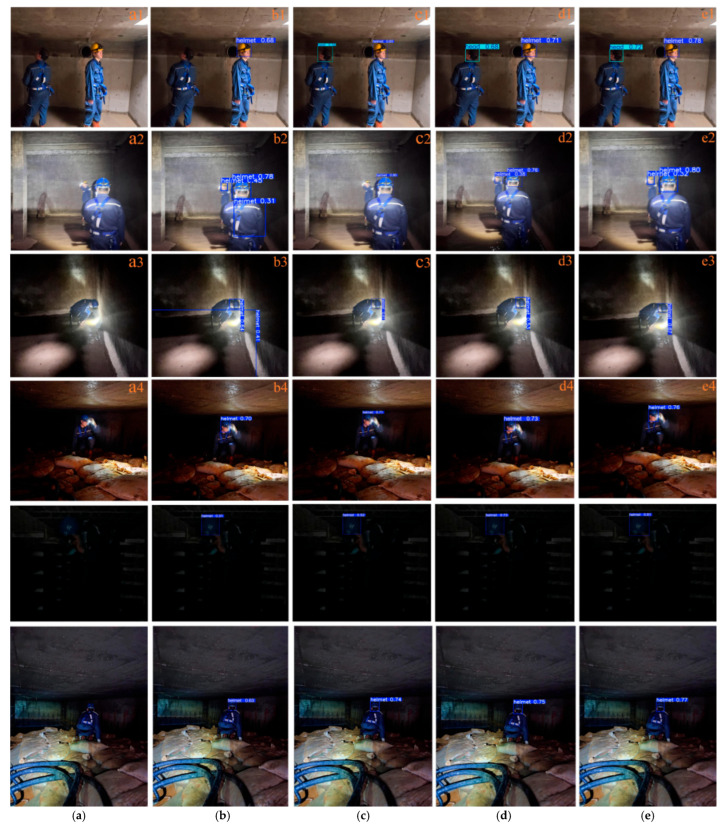
Comparison of object detection performance in environments with significant light variations: (**a**) artwork, (**b**) YOLOv8n, (**c**) YOLOv11n, (**d**) YOLOv13n, (**e**) proposed algorithm.

**Figure 11:**
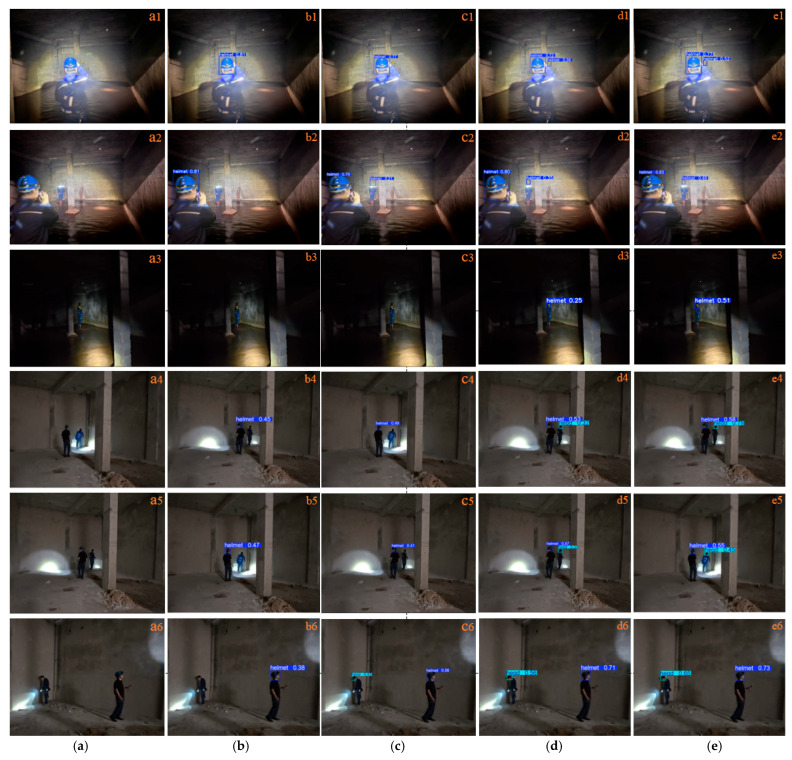
Comparative performance of small-object detection: (**a**) artwork, (**b**) YOLOv8n, (**c**) YOLOv11n, (**d**) YOLOv13n, (**e**) proposed algorithm.

**Figure 12:**
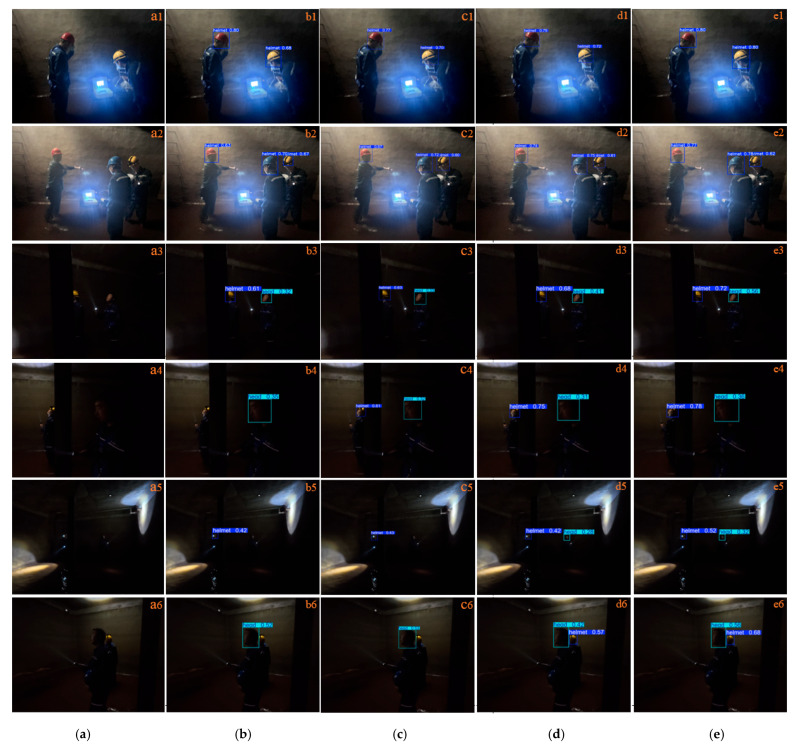
Comparison of detection performance under multi-object and occlusion conditions: (**a**) artwork, (**b**) YOLOv8n, (**c**) YOLOv11n, (**d**) YOLOv13n, (**e**) proposed algorithm.

To provide a clearer and more intuitive comparison of the superiority of the proposed improved algorithm, three representative scenarios were selected for qualitative analysis, including scenes with significant illumination variation, small-object scenarios, and multi-object scenes with occlusion. The comparison results are shown in Figure 10, Figure 11 and Figure 12.

As shown in the first group of images in Figure 10, two workers are located in an accident oil pool under natural lighting conditions. In Figure 10(b1), the YOLOv8n model fails to detect the head of one worker, resulting in a missed detection. As shown in Figure 10(b2,b3), when the fire water tank environment has no natural illumination and only headlamps and flashlights are used as light sources, the reflective strips on the work clothes and reflections from the ground cause false detections in the YOLOv8n model. Meanwhile, the detection accuracy of the YOLOv13 algorithm is slightly lower than that of the proposed method. As shown in Figure 10(a4), when workers in a cable trench turn on only their headlamps, resulting in significant illumination variation within the image, all four algorithms are able to detect the safety helmets correctly. However, the proposed method still achieves the highest detection confidence. As seen in Figure 10(a5), when workers are located inside a cable well under low-illumination conditions, the confidence scores of algorithms such as YOLOv8n are relatively low, whereas the proposed method maintains higher confidence.

Figure 11(a1,a2) show scenes containing two targets with significantly different sizes, one of which is a small object. Due to the small scale of the target, the YOLOv8n model in Figure 11(b2) fails to detect the small object. As shown in Figure 11(b3), when only a single small object is present, both YOLOv8n and YOLOv11n fail to detect the target. In Figure 11(b4–b6), the YOLOv8n model consistently fails to detect small-scale targets. Moreover, as shown in Figure 11(d4–d6), the detection accuracy of YOLOv13n is lower than that of the proposed method.

Figure 12(b1,c1,d1,e1), as well as Figure 12(b2,c2,d2,e2), illustrate that the proposed method achieves higher detection confidence compared with YOLOv8n, YOLOv11n, and YOLOv13n. As shown in Figure 12(b3,c3,d3,e3), when the targets are partially occluded, all four algorithms can detect the objects; however, the proposed method still produces the highest confidence scores. As shown in Figure 12(b4–b6), when low illumination and occlusion occur simultaneously, the YOLOv8n model fails to detect the target, resulting in missed detections.

Overall, the qualitative results demonstrate that the proposed method achieves more robust detection performance in complex scenarios, including low-illumination environments, small-object detection, and partially occluded targets.

In summary, YOLOv8n exhibits limited performance in complex power station confined space environments, particularly under conditions of drastic illumination variation, small-target missed detection, and the coexistence of occlusion and multiple targets. To address these challenges, the proposed method significantly improves detection performance through three key designs. First, the GCA-C2f module is introduced to enhance feature extraction capability under varying illumination conditions while reducing the number of model parameters, thereby improving detection robustness. Second, the I-ECA mechanism is embedded into the neck network to strengthen the model’s focus on critical targets in occluded regions, leading to improved detection accuracy under occlusion. Finally, a P2 small-object detection branch is added and combined with the WIoU loss function to optimize perception and localization stability for extremely small targets. Experimental results demonstrate that the proposed the model achieves higher detection confidence and more stable performance in various challenging scenarios, including complex illumination, dense small targets, and frequent occlusion. Overall, the method significantly enhances safety helmet detection performance in power station confined space environments.

## 4. Discussion

The ablation experiments demonstrate that each proposed component contributes to the overall performance improvement of the detection model. To better understand their roles, the contributions of each module are further discussed from the perspectives of feature representation, computational efficiency, and detection capability.

First, the proposed GCA-C2f module significantly improves computational efficiency while maintaining feature extraction capability. By replacing standard convolutions in the Bottleneck blocks with GhostConv operations, redundant feature generation is reduced and the number of parameters is significantly decreased. At the same time, the additional GhostConv layer after feature concatenation improves feature fusion efficiency, allowing the model to maintain effective feature representation under reduced computational cost. Furthermore, the introduction of the CBAM attention mechanism enhances the model’s ability to focus on informative spatial and channel features. As a result, GCA-C2f achieves a favorable balance between lightweight design and feature extraction performance.

Second, the proposed I-ECA mechanism further enhances channel feature representation. Unlike the standard ECA module, the proposed I-ECA mechanism introduces a lightweight feature expansion strategy before generating channel attention weights. This design enriches channel interaction information and allows the model to capture more discriminative features under challenging conditions such as occlusion and low contrast. Consequently, the model can better distinguish safety helmets from complex backgrounds in electric power station confined space environments.

Third, the introduction of the P2 detection branch improves the detection capability for small targets. In practical confined space scenarios, safety helmets often occupy a small portion of the image due to camera distance or perspective effects. The additional P2 detection layer enables the model to utilize higher-resolution feature maps during prediction, which enhances the sensitivity to small objects and reduces missed detections.

Finally, the adoption of the WIoU loss function improves bounding box regression during training. Compared with the original IoU-based loss functions, WIoU dynamically adjusts the gradient contribution of different samples, which helps stabilize the training process and improves localization accuracy. Although the loss function does not affect the inference complexity of the model, it leads to better convergence behavior and improved detection performance.

Overall, the proposed modules complement each other within the YOLOv8 framework. The GCA-C2f module improves computational efficiency, the I-ECA mechanism enhances feature representation, the P2 detection branch strengthens small-object detection capability, and the WIoU loss function improves localization optimization during training. The integration of these components enables the proposed model to achieve both high detection accuracy and efficient computation, making it suitable for real-time safety helmet detection in electric power station confined space environments.

## 5. Conclusions

To address the challenges of significant illumination variation and small-object detection in power station confined space environments, an improved algorithm based on YOLOv8 is proposed for safety helmet detection. Through extensive experiments, the following conclusions are obtained:

(1) The proposed GCA-C2f module integrates GhostConv and the CBAM attention mechanism. While significantly reducing the number of parameters and computational complexity, it enhances feature extraction capability under complex illumination conditions, achieving a balance between lightweight design and high detection accuracy.
(2) An improved channel attention mechanism (I-ECA) is introduced into the neck network. By suppressing background interference and irrelevant feature responses, the model’s robustness under abrupt illumination changes and target occlusion is effectively improved, thereby reducing false detections and missed detections.
(3) A P2 small-object detection branch is added to enhance sensitivity to small targets by leveraging shallow high-resolution features, significantly improving the recall rate of small-sized safety helmets. Furthermore, the WIoU loss function is adopted to optimize localization accuracy and regression stability in low-illumination and blurred scenarios, which further reduces missed detections and accelerates model convergence.
(4) Comparative experimental results demonstrate that, compared with the baseline YOLOv8n model, the proposed method improves mAP@0.5 by 2.9 percentage points and achieves superior detection performance in small-target and significant illumination variation scenarios. Meanwhile, the reduced parameter scale ensures real-time detection capability. Considering the common characteristics of confined space operations—such as enclosed structures, complex lighting conditions, and limited visibility—the proposed algorithm is not only applicable to power station-related confined spaces (e.g., cable wells and fire water tanks) but can also be extended to other industries, including mines, underground utility tunnels, and tunnels.

In future practical applications, low-light imaging devices such as night-vision cameras can be integrated to enhance image acquisition capability under weak or no illumination conditions, mitigating the adverse effects of complex lighting environments and further improving detection accuracy and environmental adaptability in power station confined space scenarios. In addition, the current framework mainly focuses on safety helmet detection. Future work will consider extending the proposed method to detect multiple types of personal protective equipment (PPE), such as safety helmets, protective clothing, and other required protective gear in power station environments, enabling more comprehensive safety monitoring for power station operation personnel.

All abbreviations used in this paper are listed in the following table.

## Figures and Tables

**Table 1:** Training parameters.

Hyperparameter	Value
Input image size	640 × 640 pixels (RGB three-channel)
Number of training epochs	100
Batch size	16
Initial learning rate	0.01
Weight decay	0.0005
Optimizer	SGD
Momentum	0.937
Warmup epochs	3
Warmup momentum	0.8
Learning rate schedule	Cosine decay

**Table 2:** Confined space safety helmet dataset description.

Class	Training Set	Test Set	Total
Targets with Significant Illumination Variation	962	231	1193
Small Targets	737	172	909
Low-Illumination Targets	1303	301	1604
Occluded Targets	998	296	1294
Total	4000	1000	5000

**Table 3:** Ablation experiments.

GCA-C2f	I-ECA	P2 Branch	WIoU	P%	R%	mAP@0.5%	mAP@0.5–0.95%	Params/10^6^	FPS	GFLOPs
				89.2	78.6	88.3	54.4	3.01	89.4	8.2
✓				88.7	78.3	87.6	54.3	2.03	101.6	6.7
	✓			89.3	79.2	89.1	56.1	3.09	88.6	8.4
		✓		89.5	78.8	88.7	55.9	3.20	83.7	9.2
			✓	89.6	80.1	88.6	55.8	3.01	89.4	8.2
✓	✓			89.8	79.6	89.7	56.8	2.11	98.3	7.0
✓	✓	✓		90.1	79.9	90.6	56.9	2.30	92.6	7.8
✓	✓	✓	✓	90.1	80.5	91.2	57.1	2.30	92.6	7.8

**Table 4:** Comparative experiments on target detection algorithms.

Model	P%	R%	mAP@0.5%	mAP@0.5–0.95%	Params/10^6^	FPS	GFLOPs
SSD	83.5	79.2	82.1	50.2	23.62	23.6	31.2
YOLOv3-tiny	85.2	79.8	85.3	52.3	8.68	51.3	15.0
YOLOv5n	89.7	78.4	87.1	53.1	2.50	93.9	7.7
YOLOv7-tiny	89.5	78.2	87.6	53.7	6.03	63.2	13.2
YOLOv8n	89.2	78.6	88.3	54.4	3.01	89.4	8.2
YOLOv10n	88.9	78.3	87.1	54.0	2.70	88.9	8.3
YOLOv11n	89.6	79.5	89.6	55.8	2.71	90.6	8.1
YOLOv13n	89.9	80.1	90.1	56.2	2.71	91.5	7.9
Improve-YOLOv5 [11]	89.5	79.2	89.1	55.3	7.51	25.6	27.1
GCW-YOLOv8n [25]	89.5	79.1	88.7	55.1	2.37	91.7	7.9
CGALS-YOLO [26]	88.3	78.5	86.7	54.1	2.56	96.8	7.1
YOLOv11n-DDH [27]	89.3	79.6	88.9	54.6	2.18	101.7	6.3
Ours	90.1	80.5	91.2	57.1	2.30	92.6	7.8

**Table 5:** Comparative experiments on a public dataset.

Model	P%	R%	mAP@0.5%	mAP@0.5–0.95%	Params/10^6^	FPS	GFLOPs
YOLOv8n	86.5	78.3	84.7	51.3	3.12	91.5	7.9
Ours	88.6	79.5	88.1	55.1	2.36	93.7	7.6

## Data Availability

The data presented in this study are available on request from the corresponding author.

## Abbreviations

**Abbreviation**	**Full Form**
AP	Average Precision
CBAM	Convolutional Block Attention Module
CIoU	Complete Intersection over Union
ECA	Efficient Channel Attention
EIoU	Efficient Intersection over Union
FN	False Negative
FP	False Positive
FPS	Frames Per Second
FLOPs	Floating Point Operations
GAP	Global Average Pooling
GCA-C2f	Ghost Convolution Attention-based C2f Module
GhostConv	Ghost Convolution
GFLOPs	Giga Floating Point Operations
GPU	Graphics Processing Unit
HDR	High Dynamic Range
IoU	Intersection over Union
I-ECA	Improved Efficient Channel Attention
mAP	mean Average Precision
P2	Level-2 Feature Detection Head
PPE	Personal Protective Equipment
SPPF	Spatial Pyramid Pooling Fast
TP	True Positive
WIoU	Wise Intersection over Union
YOLO	You Only Look Once
